# Hemorrhagic Giant Cell Tumor of the Occipital Skull Base: A Case Report and Literature Review

**DOI:** 10.7759/cureus.13832

**Published:** 2021-03-11

**Authors:** Cihan Kadipasaoglu, Andrew Wahba, Meenakshi B Bhattacharjee, Branko Cuglievan, Stephen A Fletcher

**Affiliations:** 1 Department of Pediatric Surgery, Division of Pediatric Neurosurgery, McGovern Medical School at The University of Texas Health Science Center at Houston, Houston, USA; 2 Division of Pediatrics and Patient Care, The University of Texas MD Anderson Cancer Center, Houston, USA; 3 Department of Neuropathology, McGovern Medical School at The University of Texas Health Science Center at Houston, Houston, USA

**Keywords:** hemorrhagic giant cell tumor, occipital skull base, osteoclastoma

## Abstract

Giant cell tumor of bone is a benign but locally aggressive osteolytic neoplasm that represents 3% to 5% of all primary bone tumors, primarily found at the epiphyses of long bones. Less than 1% are of calvarial origin. Herein, we report a rare case of a nine-year-old girl with a hemorrhagic giant cell tumor of the left occipital skull base.

## Introduction

Giant cell tumor of bone (GCTB) is a benign but locally aggressive osteolytic neoplasm that represents 3% to 5% of all primary bone tumors. These tumors most commonly present at the epiphyses of long bones, with initial symptoms of pain or swelling [1,2]. GCTB tends to grow rapidly and destroy its primary site and, in some instances, even metastasize. Metastases mostly occur locally near the area of the primary tumor, and if distant metastasis does occur, it typically involves the lung (approximately 3% of all GCTBs have pulmonary metastases within three to five years) [1].

Only 0.7% to 1% of GCTB cases originate in the skull; of these, the majority arise from sphenoid or temporal bone [2-6]. The preference for the sphenoid and petrous temporal bone is thought to relate to the endochondral histogenesis of these tumors, in contrast to the intramembranous ossification of the other cranial bones [7,8]. The occurrence of GCTB skull-based tumors in pre-pubertal individuals is also quite rare, as GCTB incidence peaks in adults during their third to fourth decades of life, more frequently in females [1,2,8,9].

Here we present the case of a nine-year-old girl who presented with a GCTB of the posterior fossa. We review her presenting symptoms, imaging features, treatment, and tumor histopathology, as well as the relevant literature of these rare tumors [2,5-7,9-14]. This is the third known literature report of an occipital GCTB in a pediatric patient with GCTB [9].

## Case presentation

History and examination

The patient, a nine-year-old girl with a medical history of eczema and asthma, presented to her primary care physician with a complaint of three months of worsening occipital headaches, blurry vision, balance difficulties, and falls. There was no associated nausea or vomiting and no recent illness, travel, or contact with sick people. The patient was referred to an ophthalmologist who noted bilateral disc edema and referred the patient for neurosurgical care. Neurological examination revealed normal cognition, a mild right-sided dysmetria during finger-to-nose testing, and unsteady gait during heel-to-toe walking.

**Figure 1:**
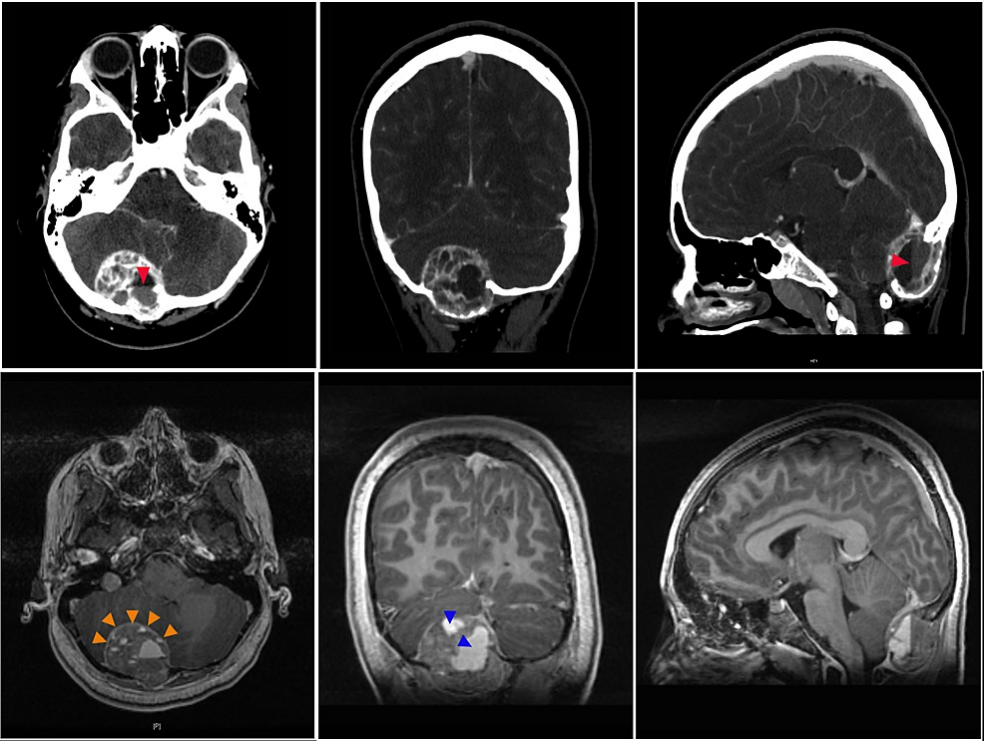
CT and MRI Brain Demonstrating Giant Cell Tumor of Right Occipital Bone Computed tomography and magnetic resonance imaging with contrast (top and bottom rows, respectively) demonstrate a 3.6 x 4.7 x 3.4 cm expansile lesion arising from the diploic spaces of the right occipital bone, expanding both inner and outer cortex and containing multiple loculations with blood-fluid levels (red arrowhead). Solid, soft tissue components avidly enhance with contrast (green arrowhead). There is mass effect on the right cerebellum without parenchymal involvement (a clear cerebral spinal fluid plane is visible, orange arrowhead) and mild effacement of the fourth ventricle without ventriculomegaly. The lesion extends to the inferior margin of the torcula and abuts the sagittal and proximal right transverse sinuses without stenosis or invasion.

Computed tomography (CT) of the brain revealed a 3.6 x 4.7 x 3.4 cm expansile, right occipital calvarial lesion with thinning of the inner and outer tables (Figure 1, top row). The lesion was heterogeneous, containing hyper-dense solid components as well as multiple cystic components with blood-fluid levels. There was mass effect on the right cerebellum and mild effacement of the fourth ventricle noted, without associated hydrocephalus. To better characterize the lesion, magnetic resonance imaging (MRI) was obtained demonstrating multiloculated, hemorrhagic and intensely enhancing soft tissue mass arising from the diploic spaces of the right occipital bone. Multiple small loculations contained hematocrit levels (Figure 1, bottom row). There was a clear cerebral spinal fluid (CSF) plane between the lesion and the cortex, with no associated edema and no venous sinus involvement. MRI of the spine was normal. 

Operation

A suboccipital craniotomy was performed, with exposure from skull base to C2. The tumor was noted to have eroded into the overlying bone, leaving only a thin superficial layer. Gross total resection of the tumor was performed, with circumferential removal of bone until normal bone was encountered. En bloc resection of the tumor mass was achieved without dural violation.

Pathology

**Figure 2:**
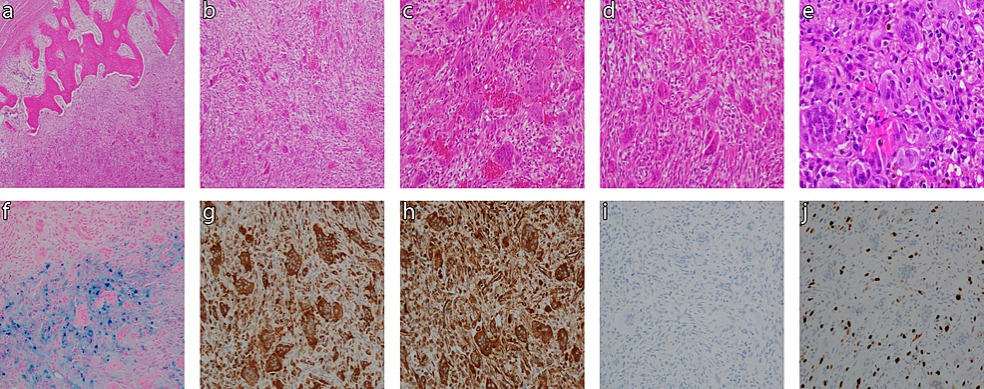
Pathology Findings from Intra-operative Tumor Specimens Intra-operative tumor sections analyzed via hematoxylin and eosin (H&E) (top row) and special stain (bottom row). Top Row: 2A Edge of tumor interface with skull bone (40x magnification). 2B Giant cell tumor of bone (100x). 2C Osteoclast-type giant cells and mononuclear spindle cells (200x). 2D-E Hemorrhagic area of tumor (200x and 400x, respectively). Bottom Row (all stains at 200x magnification): 2F Perl’s iron stain highlights aggregates of hemosiderin deposition. 2G CD68 immunostaining highlights osteoclastic giant cells and mononuclear dendritic-type stromal cells. 2H Tumor stains positive for vimentin. 2I Tumor stains negative for p53. 2J Ki67 proliferation index of 20-30%.

Tumor specimens obtained intra-operatively were sent to our institution’s pathology department for further analysis. On gross examination, the tumor appeared as an aggregate of pink-red, variegated, granular, hemorrhagic and dusky soft tissue admixed with tan-white firm bone. No obvious residual brain parenchyma was grossly identified. Serial sectioning revealed a tan-pink, focally hemorrhagic, friable cut surface (Figure 2A, 2B).

On microscopic examination, bone was found to be replaced by tumor with a relatively uniform distribution of osteoclast-type multinucleated giant cells with intervening mononuclear stromal spindle cell populations (arranged in short fascicles and storiform patterns) (Figure 2C). Areas of hemorrhage and cystic change were also noted (Figure 2D, 2E), as well as multifocal aggregates of hemosiderin deposition that were highlighted with an iron stain (Figure 2F). CD68 staining highlighted osteoclastic giant cells and mononuclear dendritic-type stromal cells (Figure 2G). Immunostaining results were positive for vimentin and negative for p53 (Figure 2H, 2I). The Ki67 proliferation index was around 20% to 30% (Figure 2J). There was no appreciable cytologic atypia, necrosis, significant increase in mitoses, or osteoid deposition within the tumor. Taken together, these findings were consistent with a GCTB.

Postoperative course

**Figure 3:**
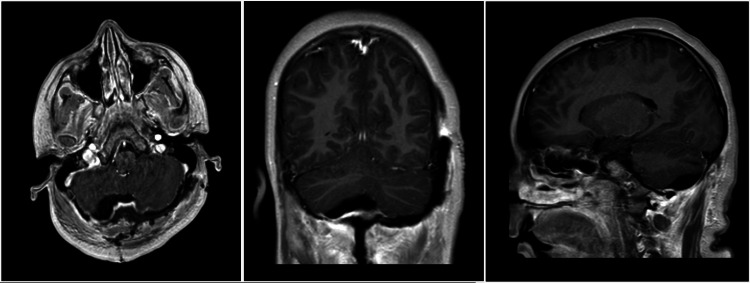
Post-operative MRI Postoperative magnetic resonance imaging with contrast reveals gross total resection of the right occipital calvarial lesion.

Following tumor removal, the patient’s postoperative clinical course was unremarkable. MRI of the brain was performed on postoperative day (POD) 1, confirming gross total resection, resolution of previous mass effects and restoration of normal fourth ventricular and cisternal spaces (Figure 3). She was discharged on the third postoperative day with a normal neurologic exam and improving vision. She has been seen for multiple follow-up visits in our clinic and remains at her neurological baseline without deficit. At six months a postoperative MRI revealed no mass and she remained without complaint.

## Discussion

Background

Hemorrhagic skull lesions are rare in the pediatric population [15]. The differential for benign hemorrhagic skull lesions includes aneurysmal bone cysts, intraosseous hemangiomas, and osteoid osteomas. Aneurysmal bone cysts typically present during the first or second decade of life and are painful to the touch, allowing for clinical examination to aid in their diagnosis. Osteoid osteomas, which account for 10% to 12% of osseous tumors in pediatric patients, are typically smaller in size (<1.5 cm, distinguishing them from osteoblastomas) and are more commonly found in the posterior elements of the cervical spine [15]. In contrast, intraosseous hemangiomas are painless and can present as enlarging skull masses, thus requiring biopsy specimens for differentiation. Among malignant skull lesions, osteosarcomas can present at the skull base with hemorrhage but are more commonly seen in patients in their third to fifth decades of life. Additional neoplasms of the occipital skull include giant cell reparative granulomas, Ewing sarcoma, brown tumor of hyperparathyroidism, chondrodysplasia, eosinophilic granuloma, plasmacytoma, desmoplastic fibroma, fibrous dysplasia, and metastatic lesions [4,16]. Given the broad treatment spectrum associated with this diverse group of skull lesions, a low threshold should exist for obtaining histopathologic confirmation of diagnosis.

GCTBs of the occipital bone are extremely rare, more so in pediatric patients, with only a few cases described in the literature (Table 1) [2,5-7,9-14]. There were six females and three males whose ages ranged from eight to 53 years, with a mean of 21.6 years. The main presenting symptom was headache in five cases with other symptoms as neck pain, difficulty swallowing, blurry vision or localized tenderness. Four patients had total resection of the tumor and only two received postoperative radiation. The outcome was good in most of the cases.

**Table 1:** Review of Prior Literature on Giant Cell Tumors of Occipital Bone Review of the nine literature reports of giant cell tumors of bone involving the occipital skull. Our case is included in this table as the ninth report. *Total dose 50 Gy was delivered at 200 cGy/fraction postoperatively using a 6-MV linear accelerator with a 2-cm safety margin.

Author (Year)	Patient Age, years	Sex	Presenting Symptom	Surgical Outcome	Radiotherapy	Outcome (Follow Up)
Troell A et al. [14] (1930)	20	M	Unknown	Partial	No	Fair (3 years)
Arseni C [9] (1975)	8	F	Unknown	Subtotal	No	Good (7 years)
Motomochi M et al. [12] (1985)	53	M	Headache, difficulties in swallowing, and disturbance of speech	Partial	2400 rads	Good (2 years)
Henderson BT and Whitwell H [17] (1988)	16	F	Occipital Headache, vomiting, and vertigo	Total	No	Good (1.5 years)
Opitz H et al. [13] (1996)	24	M	Von Recklinghausen neurofibromatosis (NF1) and bulging mass	Subtotal	No	Recurrence
Harris AE et al. [5] (2004)	24	F	Localized tenderness	Total	No	Not reported
Lu ZH et al. [11] (2011)	19	F	Headache	Total	No	Good (1 year)
Uslu G et al. [6] (2014)	22	F	Headache and neck pain	Subtotal	5000 cGy (200 cGy × 25 fractions)*	Good (1.5 years)
Present case (2020)	9	F	Headache and blurry vision	Total	No	Good (0.5 years)

The youngest pediatric patient was reported by Arseni et al., who described an eight-year-old girl with an occipital skull GCTB [9], and Henderson and Whitwell reported a right-sided occipital GCTB affecting the posterior fossa in a 16-year-old girl [17]. A recent systematic review of the GCTB literature reported only 110 known cases of GCTB localized to the skull (<2% of all reported GCTBs), of which only six patients (5% of the 110 cases) were found to have the tumor localized in the occipital skull. The youngest patient of this cohort was 19 years old [2,11,14]. Therefore, the occurrence of this tumor in a young, skeletally immature patient is exceedingly rare.

Presentation and management

The clinical presentation of patients with GCTB of the skull typically depends on the tumor location. Within the temporal region, hearing loss (usually conductive), aural fullness, retro-auricular pain, and facial weakness are common. Within the sphenoid region, headaches, visual changes, and oculo-motor deficits may predominate. In the occipital region, the presenting symptoms reported have included occipital headaches and posterior fossa symptoms (e.g. dysphagia, dysarthria, emesis) [7,12]. Given the location of tumor development in the patient described in this report, neurological deficits consistent with mass effect on the underlying cerebellar regions are not unexpected.

Current treatment options include observation or gross/subtotal resection with or without adjuvant radiation therapy. Gross total resection is the treatment of choice [2,7,18,19]. If compression occurs on critical structures (e.g. the fourth ventricle) the patient may present more emergently (e.g. acute hydrocephalus) and require immediate intervention (e.g. cerebrospinal fluid, CSF, diversion). Recurrence rates of these locally aggressive tumors are correlated with the degree of local excision [4]. After total resection, local control is achieved in 85% to 90% of all cases. In our patient, given the lesion’s clear CSF boundaries and absence of parenchymal and venous sinus involvement, gross total resection was achieved. We additionally removed surrounding, normal bone to further minimize the risk of recurrence. At 14 months after gross total resection, no recurrence was evident on repeat imaging, and the patient remains neurologically intact.

In patients who undergo subtotal resections, there is a higher rate of recurrence (40% to 60%) within two years [5]. In such cases, where gross total resection cannot be achieved, adjuvant therapy has been employed [4]. However, there is not yet enough evidence to determine the long-term efficacy and side effects of radio-/chemo-therapy [2,4,7]. The monoclonal antibody denosumab has also been employed because of its inhibition of osteoclast-like cell function and recruitment. However, its usage is currently preserved for patients who are not good surgical candidates or patients with advanced unresectable or recurrent tumors, as it carries a known risk of jaw osteonecrosis [20].

## Conclusions

Giant cell tumors are typically benign but locally aggressive. Literature reports of these tumors are scarce and are usually confined to case reports and series. This case advances the literature by providing a rare instance of a GCTB localized to the occipital bone of the posterior fossa in a pediatric patient and highlights the importance of considering GCTB in the differential diagnosis of patients with cranial bone neoplasms. In all cases, gross total resection is the treatment of choice, as the extent of surgical resection has been shown to be predictive of prognosis. If gross total resection is not possible, subtotal resection with adjuvant therapy can be employed. However, more studies are required to further investigate the efficacy of these alternative treatment strategies.
